# Study protocol for a cluster randomised controlled feasibility trial evaluating personalised care planning for older people with frailty: PROSPER V2 27/11/18

**DOI:** 10.1186/s40814-020-00598-x

**Published:** 2020-04-28

**Authors:** Anne Heaven, Peter Bower, Bonnie Cundill, Amanda Farrin, Marilyn Foster, Robbie Foy, Suzanne Hartley, Rebecca Hawkins, Claire Hulme, Sara Humphrey, Rebecca Lawton, Catriona Parker, Neil Pendleton, Robert West, John Young, Andrew Clegg

**Affiliations:** 1 Academic Unit for Ageing and Stroke Research, University of Leeds, Bradford Institute for Health Research, Bradford Teaching Hospitals NHS Foundation Trust, Bradford, BD9 6RJ UK; 2 NIHR Older People and Frailty Policy Research Unit, Centre for Primary Care and Health Services Research, Manchester Academic Health Science Centre, University of Manchester, Manchester, M13 9PL UK; 3 Leeds Institute of Clinical Trials Research (LICTR), Clinical Trials Research Unit, University of Leeds, Leeds, LS2 9JT UK; 4 Leeds Institute of Health Sciences, School of Medicine, University of Leeds, Leeds, LS2 9JT UK; 5 Health Economics Group, Institute of Health Research, University of Exeter, Exeter, LU EX1 2 UK; 6 Speciality Clinical Lead Older People Bradford District & Craven CCGs Scorex House West, 1 Bolton Rd, Bradford, BD1 4AS UK; 7 School of Psychology, University of Leeds, Leeds, LS2 9JT UK; 8 University of Manchester, Salford Royal Hospitals NHS Trust, Stott Lane, Salford, Greater Manchester M6 8HD UK

**Keywords:** Personalised care planning, Quality of life, Older people, Frailty, eFI, Cluster, RCT

## Abstract

**Background:**

Frailty is characterised by increased vulnerability to falls, disability, hospitalisation and care home admission. However, it is relatively reversible in the early stages. Older people living with frailty often have multiple health and social issues which are difficult to address but could benefit from proactive, person-centred care. Personalised care planning aims to improve outcomes through better self-management, care coordination and access to community resources.

**Methods:**

This feasibility cluster randomised controlled trial aims to recruit 400 participants from 11 general practice clusters across Bradford and Leeds in the north of England. Eligible patients will be aged over 65 with an electronic frailty index score of 0.21 (identified via their electronic health record), living in their own homes, without severe cognitive impairment and not in receipt of end of life care. After screening for eligible patients, a restricted 1:1 cluster-level randomisation will be used to allocate practices to the PROSPER intervention, which will be delivered over 12 weeks by a personal independence co-ordinator worker, or usual care. Following initial consent, participants will complete a baseline questionnaire in their own home including measures of health-related quality of life, activities of daily living, depression and health and social care resource use. Follow-up will be at six and 12 months. Feasibility outcomes relate to progression criteria based around recruitment, intervention delivery, retention and follow-up. An embedded process evaluation will contribute to iterative intervention optimisation and logic model development by examining staff training, intervention implementation and contextual factors influencing delivery and uptake of the intervention.

**Discussion:**

Whilst personalised care planning can improve outcomes in long-term conditions, implementation in routine settings is poor. We will evaluate the feasibility of conducting a cluster randomised controlled trial of personalised care planning in a community population based on frailty status. Key objectives will be to test fidelity of trial design, gather data to refine sample size calculation for the planned definitive trial, optimise data collection processes and optimise the intervention including training and delivery.

**Trial registration:**

ISRCTN12363970 – 08/11/18.

## Background and rationale

Frailty is a condition characterised by reduced biological reserves and increased vulnerability to adverse outcomes [1]. Frailty has characteristics of considerable clinical importance, including higher reversibility at early stages than disability and higher predictive value than chronic disease for adverse outcomes [2]. Single disease management frameworks and targets are less relevant for older people living with frailty, who frequently have multi-morbidities, personal predicaments and social problems [3]. Current best practice suggests that care for older people with frailty should be proactive and person-centred, responsive to personal experiences of illness, individual priorities and predicaments [1, 4, 5].

Personalised care planning (PCP) is a promising way to achieve this shift towards proactive, person-centred care in frailty [4–6]. It is an anticipatory, negotiated series of guided conversations between a patient and a suitably trained individual to clarify goals, options and preferences and develop an agreed plan of action [4]. The process aims to ensure that individual values and concerns shape how care is provided, instead of a focus on individual disease management. In PCP, shared decision-making is a crucial mechanism, involving a collaborative discussion of management goals (goal setting) and developing an agreed plan for achieving these goals (action planning). Shared decision-making enables linkage to additional mechanisms for improving outcomes through more effective self-management, better care coordination and better access to community resources (social support) [4].

A 2015 Cochrane review summarised evidence from 16 international randomised controlled trials (RCTs) and identified that PCP for long-term conditions (LCTs) can improve physical and mental health and self-management capability. Effects appear greater when PCP is more comprehensive and integrated into routine care [4]. However, the majority of studies focused on single LTCs; no studies were identified that selected participants on the basis of frailty. Furthermore, a comprehensive evaluation of PCP in the UK has identified widespread, poor implementation [7, 8].

In the UK, the NHS long-term plan sets out the blueprint and key ambitions for the health service over the next 10 years [9]. The plan includes a focus on establishing primary care-based multidisciplinary teams to provide tailored support for older people living with frailty to enable them to live independently at home for longer. It is therefore essential that promising interventions, such as PCP, are designed to be sufficiently robust and flexible to facilitate integration with commissioning and provider organisations, prior to rigorous definitive trial evaluation. Age UK, the largest voluntary sector organisation representing older people in the UK, developed an integrated personalised care planning (IPCP) service in partnership with older people, their families and carers. Although the service was piloted, further research is required to optimise the service and evaluate its effectiveness. We have collaborated with the Age UK National programme to optimise the IPCP service and accurately identify those who are most likely to benefit from the PCP based on their level of frailty and health and social care use. The result is the Personalised Care Planning for Older People (PROSPER) intervention, designed to improve quality of life for older people with frailty and reduce use of health and social care services.

Here, we describe the protocol for the feasibility trial evaluation of PROSPER compared with usual care (UC) to inform the design of a definitive RCT.

### Aim and objectives

The overall aim of this trial will be to assess the feasibility of conducting, and to inform the design of, a definitive RCT to investigate the clinical and cost-effectiveness of PROSPER compared with UC.

Specific objectives will be to determine:

- Appropriate methods to identify, approach and select general practices to participate in the trial.
- Appropriate methods to screen and consent potential participants to the trial, and the extent to which blinding can be maintained.
- Recruitment and follow-up rates of general practices and participants.
- How the sample size should be refined for the definitive trial.
- How data collection processes can be optimised.
- If the intervention can be further optimised, including through strategies to monitor and enhance reliability and validity of the intervention (fidelity of intervention training, delivery, receipt and enactment).
- If proposed outcome measures are feasible and acceptable to participants.

## Methods

### Trial design

PROSPER is a multi-centre, two-arm, feasibility cluster RCT. A cluster is defined as a general practice or a group of practices where the practices share significant staff and/or services, or were due to merge during the trial period. The cluster design was appropriate because the intervention was envisaged to be a multidisciplinary team-based intervention, delivered at the level of the general practice. An intervention embedded within primary care would also mean there would have been a high risk of contamination in an individually randomised trial from practice staff treating UC participants.

We also plan to embed a mixed-methods evaluation, informed by the MRC guidance for process evaluation of complex interventions [10] and the NIH Behaviour Change Consortium framework [11].

### Study setting

Participants will be recruited from 11 clusters in Bradford and Leeds, West Yorkshire. These locations cover a range of multi-ethnic rural and urban populations, so ensuring the generalisability of findings. Four of the 11 clusters will be sampled for the process evaluation.

### General practice (cluster) eligibility

General practices in Bradford and Leeds who use SystmOne, EMISWeb, Vision or Microtest primary care electronic health record systems will be eligible for inclusion. Practices with an existing or planned PCP service for older people with frailty that has significant overlap with the PROSPER model will not be eligible.

### Participant eligibility

Following agreements from the general practices to participate, all patients at a practice will be screened for eligibility by a practice administrative staff. The eligibility list will be further reviewed by a lead GP to avoid inappropriate contact being made.

Patients meeting *all* of the following criteria at time of screening will be eligible for inclusion:

- Aged 65 or over.
- Frailty defined by the electronic frailty index (eFI) [12] score ≥ 0.21.
- Willing and able to give informed consent (or provide a personal consultee).

Patients meeting *any* of the following criteria will not be eligible for inclusion:

- Resident of a care home at time of screening.
- Registration on the Gold Standards Framework (GSF) or end of life care.
- Coded diagnosis of severe dementia or a Montreal Cognitive Assessment (MoCA) score of ≤ 10 at baseline.
- Deemed inappropriate by their general practitioner (GP) (documented reason).
- Member of the household currently or previously participated in the trial.

### Carer involvement

Where the participant or their consultee has consented to the carer’s involvement, they will also be included. Carers lacking the capacity to provide written informed consent will not be included.

### General practice recruitment and randomisation

A range of approaches will be used to engage with general practices including local National Institute for Health Research Clinical Research Networks (NIHR CRNs), professional networks and practices listed on the Public Health England (PHE) website [13].

Practices will be randomised on a 1:1 basis either to implement the intervention or continue with usual care (UC) only, using an algorithm for covariate-constrained randomisation to achieve balanced allocation between the trial arms on sample size and baseline characteristics expected to be potential confounding factors [14]. Randomisation will be performed by the statistical team at the Clinical Trials Research Unit (CTRU). Following randomisation, the CTRU will inform the practice manager of the allocation to allow trial procedures to be implemented. Knowledge of the allocation will be restricted to staff in the practice multidisciplinary team (MDT) who will be involved in the delivery of the intervention. MDTs may include a variety of staff from GPs to community matrons. We will seek to maintain blinding of practice staff not directly involved in intervention delivery. Instances of un-blinding will be recorded.

### Participant recruitment/randomisation

To allow for scheduling of the intervention delivery and ensure that a similar number of participants are recruited from each general practice, eligible participants will be invited to consent to data collection in a phased approach. Following cluster randomisation, the CTRU will periodically generate a random sample of eligible participants to approach. Eligibility lists from each cluster will be ranked according to eFI to ensure a similar distribution of eFI scores that are randomly sampled across the clusters and between trial arms. For participants randomly sampled, eligibility will be re-confirmed by a practice staff prior to sending letters of invitation for trial participation (data collection). This process will be the same in both trial arms. PROSPER recruitment uses an opt-out approach, i.e. where an individual pro-actively declines to be contacted by a researcher by returning a negative expression of interest, to maximise potential for participant recruitment. This will allow researchers’ access to patient contact details after the opt-out period has ended and is aligned with previous research in this area [15, 16].

If there is no response to the invitation within 14 days, a researcher blinded to allocation will contact the potential participant by phone to discuss the study. If an individual is interested, the researcher will make an appointment to visit them. If not, the reason will be recorded, if possible. Once a participant is registered in the study, the Age UK delivery team will receive notification of those in the intervention arm and proceed to make contact. Participants in the control arm will continue to receive usual care.

**Fig. 1:**
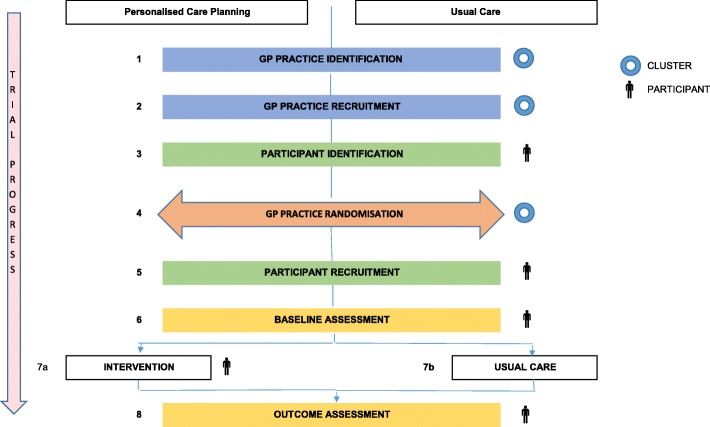
Assignment of interventions

The process for assignment of interventions is outlined in Fig. 1.

### Qualitative sample

A purposive sample will be informed by learning from previous case studies of the National Age UK IPCP and will be targeted to ensure maximum variation to support further intervention optimisation—within the resource constraints of a feasibility study. We anticipate that this will be a mixture of client/carer dyads, those living alone and from different ethnic backgrounds.

### Trial consent or assent

Participant consent, recruitment and collection of baseline data will be undertaken in the potential participant’s own home by a researcher blind to cluster allocation. Home visits will be made to reduce participant burden and facilitate the assessment of capacity and cognition. Before consent researchers will make an assessment of the patient’s capacity to consent, using the Mental Capacity Act (MCA) framework (2005) [17]. Individuals lacking capacity to consent will not be excluded from this study as they may still benefit from engagement with the intervention. If a potential participant lacks capacity to consent, they will be asked to nominate a personal consultee to advise on their behalf. If the potential participant is unable to identify a personal consultee, the GP will be requested to identify an appropriate potential personal consultee. If a personal consultee is not available, no further contact will be made.

Following participant consent or consultee assent, researchers will administer the Montreal Cognitive Assessment (MoCA). Individuals scoring less than 10 will be deemed ineligible because they would be unable to take part in the shared decision making aspects of PCP. A carer will be identified (if available) and after consent; in the intervention arm, the practice administrative staff /PIC worker will be notified which patients have been registered.

Reasons for declining involvement will be noted if provided. The right of a patient (and carer) to refuse participation without giving reasons will be respected.

### The PROSPER intervention

PROSPER is a manualised intervention delivered to participants in their own home by trained Age UK Staff—Personal Independence Co-ordinators (PICs) and support workers (SW). It is based on the social cognitive theory (SCT) which resonates closely with the tenets of PCP in the context of frailty [18]. SCT specifies factors governing the acquisition of competencies that can profoundly affect physical and emotional wellbeing [19]. It identifies knowledge, skills, self-efficacy, outcome expectations, goals and concrete plans, and the perceived social and environmental facilitators and impediments as core determinants influencing health habits. Therefore, PROSPER targets confidence building and providing older people with knowledge and skills to set personal goals, develop appropriate action plans and personalised problem-solving strategies. PIC workers will undertake ‘guided conversations’ and shared decision-making, developing goals and action plans with older people. Support workers will practically support older people to achieve their goals. Both workers will be employed by a third sector organisation but work as part of a wider multidisciplinary team, co-located within a general practice.

Key intervention materials include the intervention manual and associated training package. The manual is divided into four sections: section 1 provides an overview of PCP, while sections 2 and 3 provide a guide on how to set up and deliver the service. Section 4 describes some of the key concepts which have shaped the development of PROSPER and includes a list of references for further information. Section 5 (the toolkit) contains the necessary templates of practical tools needed for set up and delivery of the PROSPER service, e.g. job descriptions, service invitation letters and action-plans. In addition, the manual contains a list of behavioural change techniques for use with older people identified in a systematic review.

The training package uses blended learning (including on-line, face to face and experiential delivery methods) and includes background to frailty, ‘the Art of the Guided Conversation’, motivational interviewing and a summary of behaviour change techniques for use with older people. Additional motivational interviewing training sessions enable reflection on the use of this approach after initial familiarisation in the field. Intervention training will take approximately 27 h.

Core intervention activities include a guided conversation to set personal goals and develop appropriate action plans; a two-month review to reflect on progress, problem solve, and confirm appropriate next steps; and ‘Graduation’ (discharge from service). In line with these activities, a minimum of three face-to-face contacts will be made. Additional face-to-face, telephone or email contact is expected within the 12-week intervention period. The amount of additional contact will vary depending on the needs of the older person.

We will continue to optimise the intervention using an iterative approach, with a focus on staff training, contextual factors and implementation in preparation for a definitive clinical trial.

### Consent to the process evaluation

All participants will be recruited to the process evaluation by an independent researcher not involved in baseline or follow-up data collection for the feasibility trial. Consent will be sought from each participating individual separate to the trial process. Identified clients will be sent an information booklet about the process evaluation at the same time as the invitation letter and intervention service information. The PIC worker will contact participants by telephone to explain the purpose of the observations and gain verbal consent for the process evaluation researcher to attend their first visit. Carers and significant others will also be asked for consent. Definitive consent for the PE will be sought by the independent researcher at the time of the PE data collection to avoid a significant time lapse between data collection and consent. In addition, we will seek specific consent for publication of any quotes and/or voice recordings for use in dissemination. Participants have the right to withdraw at any time and may withdraw from the process evaluation but continue in the trial.

### Usual care

Practices allocated to the control arm will continue to provide usual care, defined as ‘The wide range of care that is provided in a community whether it is adequate or not, without a normative judgement’ [20]. To increase external validity and relevance of trial findings to clinical practice, we will use an unrestricted usual care approach in both control and intervention arms in line with pragmatic trial design and the expected heterogeneity of available treatments [21]. It is also anticipated that some participants will receive disease-specific care planning for single long-term conditions. Use of specific services will be recorded at baseline and follow-up assessments and documented on case report forms (CRFs) or collected via routine data.

### Trial data collection

Participant outcome measures will be collected via (blinded researcher supported) self-completion questionnaires at baseline and postal follow-up questionnaires at 6 and 12 months. Prior to sending out the follow-up questionnaires, participant survival status and address will be confirmed. Supported completion, by a blinded researcher, at follow-up will be provided if a participant loses capacity during the trial. Researchers will receive training on all study specific assessments to ensure standardised completion and to monitor participant fatigue, which may necessitate data collection over multiple days.

Baseline and follow-up assessments will be ordered to prioritise primary outcome data. Participants will also receive a small unconditional monetary incentive (£10 gift voucher) to encourage follow-up at 12 months. We will additionally seek to promote participant retention through regular newsletters.

Participants, including carers, will be free to withdraw consent at any time. Where possible, clarification on the specific area of withdrawal, e.g. access to health and social care records will be sought, and the reason for withdrawal will be recorded, but not obligatory. Previously collected data will be used in the analyses.

### Process evaluation data collection

We will use a range of methods to explore intervention implementation across four intervention practices in two geographic localities. In addition to collecting primary data via non-participant observations, interviews and questionnaires, we will make use of secondary data sources wherever possible to reduce burden, i.e. CRFs from the trial and case reports from the Age UK delivery teams. Non-participant observations will be conducted, with consent, during delivery staff training, ‘guided conversations’, two month reviews, ‘graduations’ and MDT meetings. Semi-structured interviews will take place with delivery team staff: MDT members (in both control and intervention practices) and participants and their carers. In addition, we will use the NoMAD instrument [22] to describe how delivery and MDT staff view the intervention with reference to the four constructs of the normalisation process theory [23]. This will elicit views about how the intervention potentially impacts on their work and their expectations about whether it could become routine. It will also be used to identify any potential areas of training that need to be tailored to accommodate differences in each of the stakeholder groups. All observations and interviews will be audio or video recorded as appropriate.

### Outcome measures

A summary of assessments and outcome measures can be found in Table 1. If any assessment has been completed as part of usual care for a participant, the assessment will not be repeated for that participant for the purposes of the trial in order to minimise participant burden and the potential for recall bias. 
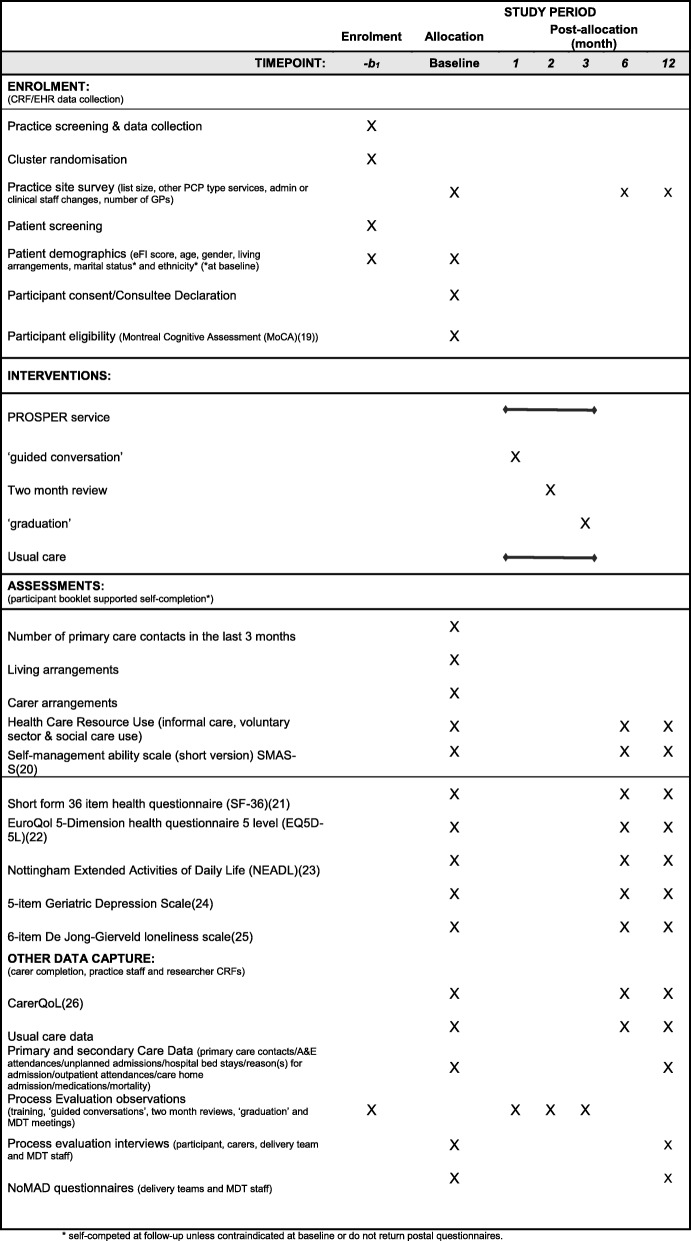

**Table 1:**
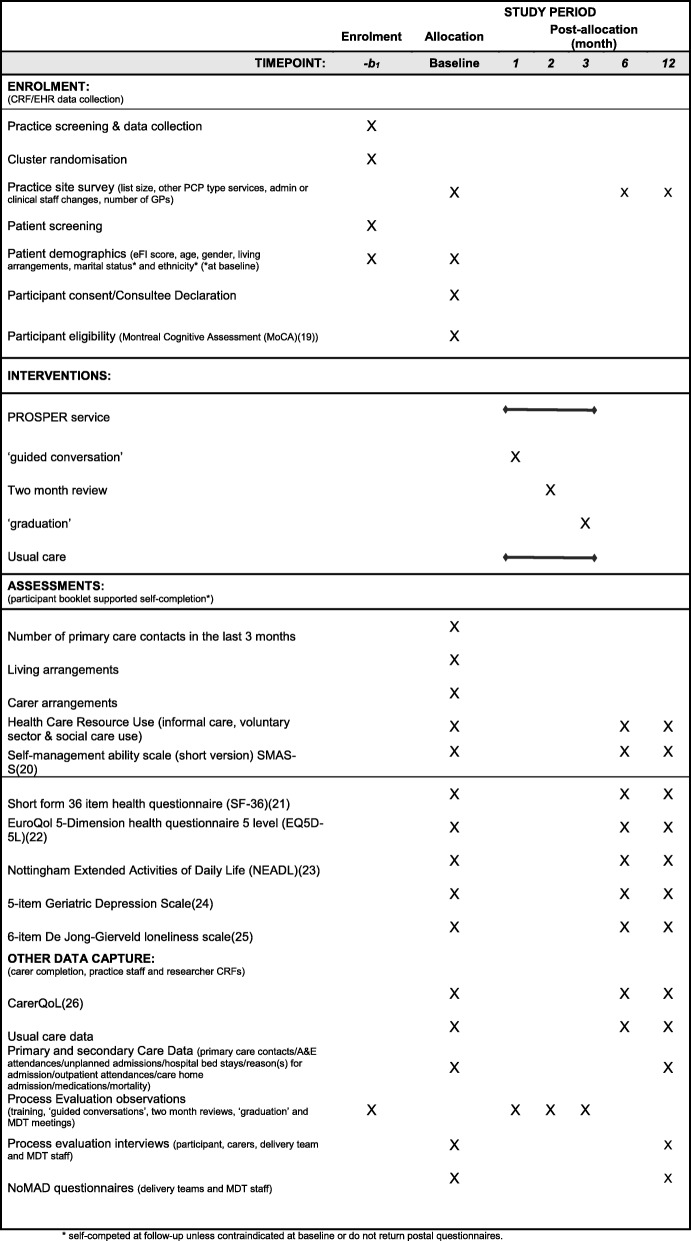
SPIRIT: summary and timing of assessments

Additional delivery team activity data such as type, number and duration of contacts will also be collected to identify the scope and reach of the intervention.

Data will be collected from practices and PIC teams during follow-up to determine if any change in practice has occurred during the study period and evaluate level of contamination.

### Sample size

Formal sample size calculations are not required for feasibility trials. However, based on PIC capacity, assuming 20% of patients will meet eligibility criteria and 33% of those will consent, we estimate an average of 25–50 patients per practice will participate in the study. A minimum of four clusters per arm is required to allow replication within study arm. We therefore aim to recruit up to 400 participants (200 per arm) from 11 clusters. A sample of this size will also allow us to estimate recruitment and follow-up rates with a maximum likely error of less than 10%. We originally planned to recruit from eight clusters in two geographic localities. However, funding was uncertain in one of the localities, and to avoid the risk of insufficient clusters, we increased the number of clusters in the funded locality. When funding became available in the second locality, the study team felt the addition of another four practices would elicit valuable contextual data for the process evaluation.

### Data management

All data will be analysed and securely stored at the CTRU. Only these data will be used in the analyses for all abstracts and publications relating to the questions posed within the study protocol—with the exception of process evaluation data. All analyses will be performed by the CTRU statistical team. All data will be stored for 10 years. Paper data will be disposed of with confidential waste, and electronic data no longer required for analysis will be deleted.

### Statistical methods

A single final analysis is planned after the trial is closed to recruitment and follow-up, and the trial database has been cleaned and locked. All analyses and data summaries will be conducted on the intention-to-treat (ITT) population [24]. Participants will be analysed according to the intervention they received. The analysis will focus on descriptive statistics and confidence interval (CI) estimation.

### Screening, recruitment and follow-up

Recruitment strategies for general practices and participants will be evaluated by summarising the screening, eligibility, consent/assent, registration/randomisation processes and follow-up in line with CONSORT guidance [25]. Reasons for non-participation will be reported alongside timing of and reasons for withdrawal by study arm to examine whether there are any systematic differences which could be attributed to the intervention.

### Intervention delivery

Baseline characteristics of the participants, practices and PIC teams will be summarised. Details of attendance at and delivery of the training will be provided to inform procedures for training of PICs and acceptability of training. Intervention delivery will be assessed in line with TiDIER [26], reporting to inform uptake, acceptability and fidelity alongside findings from the process evaluation. Usual care will be summarised across all general practices.

### Refinement of the sample size

As proposed, primary outcome measures for the definitive trial, the mean SF-12 and SF-36 Physical Component Summary (PCS), and Mental Component Summary (MCS) scores and variances will be estimated at baseline and 12-month follow-up alongside the intra-cluster correlation coefficient (ICC) and corresponding 95% confidence interval (CI). Rates of deaths, withdrawal and losses to follow-up will also inform the sample size calculations for a definitive trial.

### Feasibility and acceptability of outcome measures and methods of data collection

The number and proportion of participant completed data at baseline; 6- and 12-month follow-up will be reported by arm alongside completeness rates for individual measures. Missing data will be summarised. Participant outcome measures will be summarised at each time point by arm along with a measure of variation. A range of CIs will be constructed for differences between control and intervention arms at 12 months. No formal testing will be done on these data.

### Qualitative analysis

We will use framework analysis to collate and code interview and observation data according to our original objectives, including training, structures and adaptations. Alongside this, we will generate further inductive themes to add to the framework. The framework will be tested on a sub-set of the data and refined through discussion among the research team, before the final framework being applied to the remaining data.

The analysis aims to explore factors shaping implementation. Qualitative data, from observations, videos, interviews and documentary analysis, will also be explored for inconsistencies in delivery and deviation from the protocol. Findings will be used to inform the development of the logic model using an iterative-inductive approach to identify common themes. These will be reviewed by the Programme Management Group and Intervention Development Group, which will discuss and agree any modifications required. We will use directed content analysis to interrogate the PIC worker notes for evidence of BCT, goal setting and monitoring. Data from all sources will be triangulated to capture different dimensions and increase validity of the findings. The constructs within NPT: coherence, cognitive participation, collective action and reflexive monitoring will be employed as sensitising concepts in order to highlight areas of implementation weakness.

### Trial progression criteria

Progression criteria for a definitive RCT are based on a traffic light system of green (proceed to RCT), amber (review RCT design and/or intervention implementation) and red (stop and reconsider design and/or intervention). Progression criteria will be assessed 12-month post-randomisation within the areas of trial recruitment, intervention and follow up, as detailed in Table 2. 
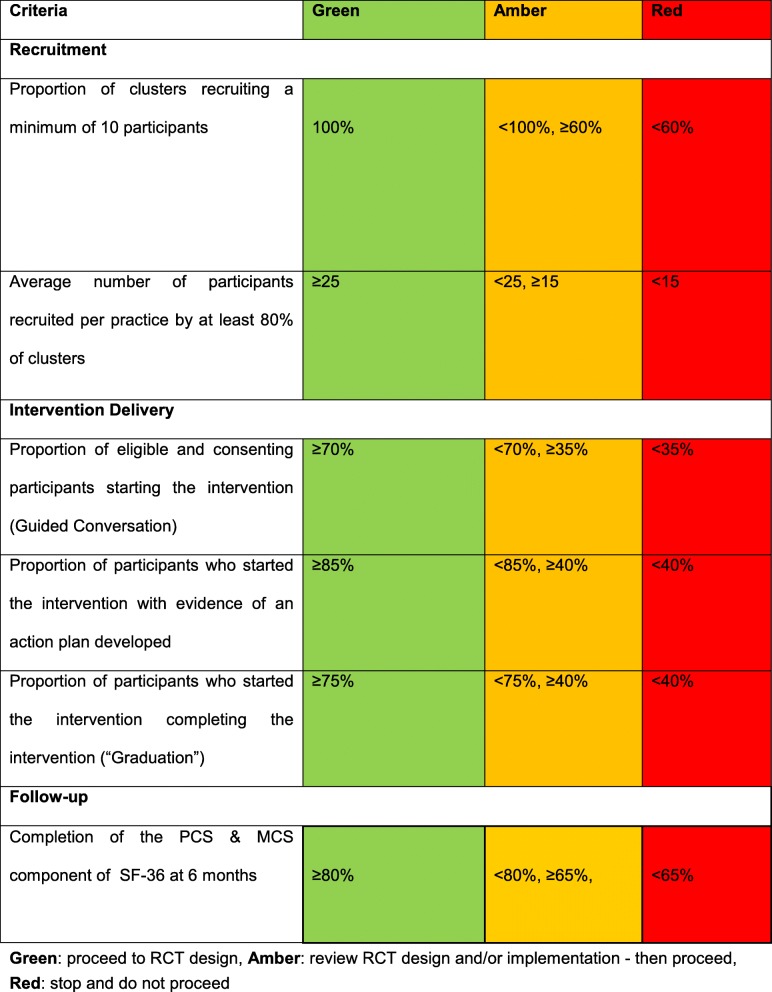

**Table 2:**
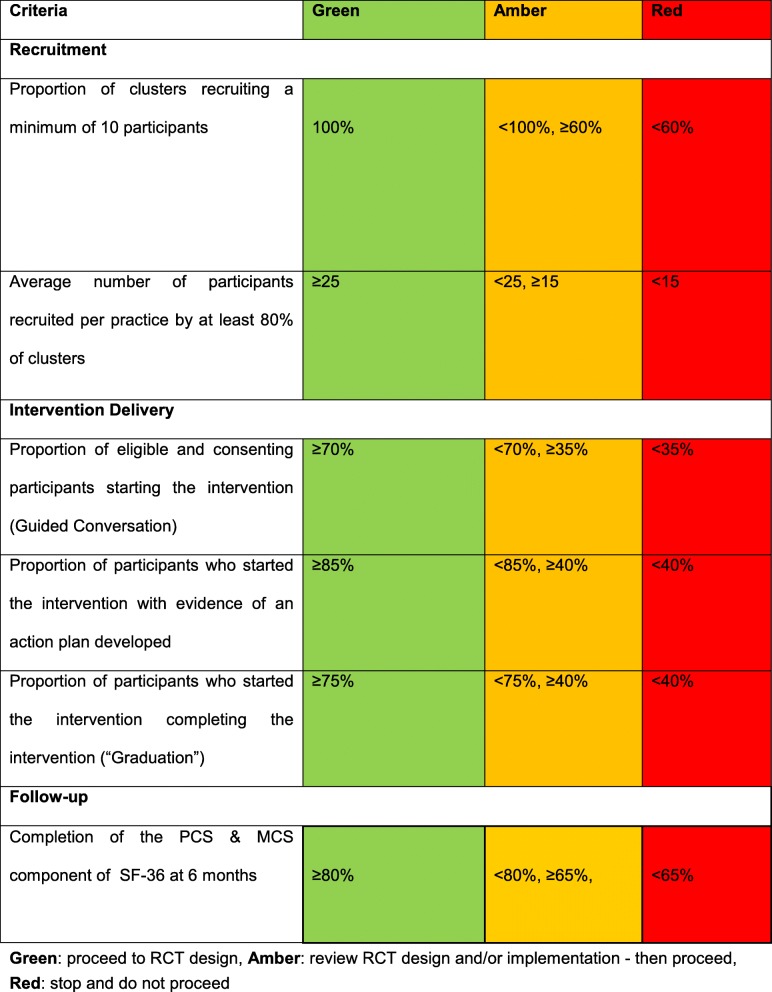
Progression criteria

### Trial organisation and governance

#### Data monitoring

Data will be monitored for quality and completeness by the Clinical Trials Research Unit (CTRU), using established verification, validation and checking processes. The Trial Management Group (TMG), comprising the chief investigator (CI), CTRU team, other key co-applicants and a GP representative will be assigned responsibility for the clinical set-up, on-going management, promotion of the trial and for the interpretation and publishing of the results. The TMG will be responsible for auditing consent procedures, data collection, trial end-point validation and database development.

The TMG will report to the Trial Steering Committee (TSC) bi-annually. The TSC will comprise an independent chair, health economist and statistician.

#### Missing data

Missing data, except individual data items collected via the postal questionnaires, will be chased until they are received or confirmed as not available. Reminders will be sent to participants if postal questionnaires are not returned on time. Source data verification exercises on a sample of participants may be carried out by staff from the CTRU/Sponsor.

#### Harms

In this patient population of older people with frailty, there is potential for acute illness resulting in hospitalisation, new medical problems and deterioration of existing medical problems. In recognition of this, events fulfilling the definition of an Adverse Event (AE) or Serious Adverse Event (SAE) will not be reportable in this study unless specified or fulfil the definition of a Related and Unexpected Serious Adverse Event (RUSAE). All RUSAEs will be reviewed by the CI and will be subject to expedited reporting to the sponsor and the main Research Ethics Committee (REC) by the CTRU on behalf of the CI within 15 days.

## Discussion

The PROSPER feasibility trial will provide novel evidence on the feasibility of a cluster RCT evaluation of a PCP intervention for older people living with frailty. The study will address key methodological design considerations, including fidelity of trial design and estimates to enable definitive trial sample size calculation. Generated evidence will also inform how data collection methods should be optimised for trials recruiting older people with frailty, and so will provide important information for clinical trial design internationally. Alongside these elements, the feasibility trial will study how the intervention should be optimised in terms of training, delivery, receipt and enactment as key fidelity considerations.

Informing design and implementation of a robust definitive trail is critically important, given the focus on transformation of services for older people living with frailty based on best available evidence locally in Leeds and Bradford, more widely across the UK as part of the NHS long-term plan, and internationally.

## Data Availability

Any data sharing requests should be sent to the corresponding author and would be subject to review and approval by the TSC and require data sharing agreements.

## Abbreviations

AE: Adverse event
CI: Confidence interval
CRN: Clinical Research Network
cRCT: Cluster randomised control trial
CRF: Case report form
CTRU: Clinical Trials Research Unit
eFI: Electronic frailty index
EQ5D: 5-level EQ-5D
GDS: Geriatric Depression Scale
GDPR: General Data Protection Regulations
GP: General practitioner
HES: Health Episode Statistics
ICC: Intraclass correlation coefficient
ICF: Individual consent form
ISRCTN: International Standard Randomised Controlled Trials Number
IRAS: Integrated Research Application System
ITT: Intention to treat analysis
LICTR: Leeds Institute of Clinical Trials Research
LTC: Long-term condition
LICTR: Leeds Institute of Clinical Trials Research
LIHS: Leeds Institute of Health Sciences
MCA: Mental Capacity Act
MCS: Mental Component Summary
MoCA: Montreal Cognitive Assessment
MDT: Multidisciplinary team
NEADL: Nottingham Extended Activities of Daily Living
NHS: National Health Service
NIHR: National Institute of Health Research
PCP: Personalised care planning
IPCP: Integrated personalised care planning
PCS: Physical Component Summary
PHE: Public Health England
PIC: Personal Independence Coordinator
PMG: Programme Management Group
PSC: Programme Steering Committee
QoL: Quality of life
RCT: Randomised controlled trial
RUSAE: Related Unexpected Serious Adverse Event
SAE: Serious adverse event
SCT: Social cognitive theory
SF-36: Short form 36-item health questionnaire
SW: Support worker
TMG: Trial Management Group
TSC: Trial Steering Committee
UC: Usual care
UK: United Kingdom
